# Rhinologic Procedures in the Era of COVID-19: Health-care Provider Protection Protocol

**DOI:** 10.1177/1945892420927178

**Published:** 2020-05-14

**Authors:** Mohamed A. Taha, Christian A. Hall, Richard F. Rathbone, Luke A. Corsten, Charles R. Bowie, Paul J. Waguespack, Richard Stanger, Megan M. Stevenson, Brittany A. Zito, Henry P. Barham

**Affiliations:** 1 Rhinology and Skull Base Research Group, Baton Rouge General Medical Center, Baton Rouge, Louisiana; 2 Department of Otorhinolaryngology, Cairo University, Cairo, Egypt; 3 Sinus and Nasal Specialists of Louisiana, Baton Rouge, Louisiana; 4 The NeuroMedical Center, Baton Rouge, Louisiana

**Keywords:** endoscopic sinus surgery, skull base, endoscopy, rhinology, viral transmission

## Abstract

**Introduction:**

SARS-CoV-2 has been identified as the pathogen causing the outbreak of Coronavirus Disease 2019 (COVID-19) that started in Wuhan, China, in December 2019. SARS-CoV-2 has human-to-human transmission ability and universally contagious to all populations. The main transmission patterns are respiratory droplets transmission and contact transmission. The purpose of this study is to propose a protocol that may be used as a guide to reduce the incidence of COVID-19 infections among otolaryngology care teams.

**Methods:**

A prospective cohort study was conducted to show the efficacy of our protocol to prevent transmission to health-care providers from March 11, 2020 through April 14, 2020. The protocol consisted of a series of protective measures that we applied to all health-care providers, then testing of our providers for COVID-19 using reverse transcription polymerase chain reaction along with immunoglobulin M (IgM) and immunoglobulin G (IgG) testing at the end of the study period to ensure effectiveness.

**Results:**

Our protocol resulted in zero transmissions to our health-care providers during the duration of the initial study. We were involved in greater than 150 sinonasal, skull base, open airway, and endoscopy procedures during this study. At the conclusion of the initial 5 weeks, we had no health-care providers test positive for SARS-CoV-2.

**Conclusion:**

According to our proposed protocol, we were able to provide care for all patients in clinic, hospital, emergent, intensive, and surgical settings with no transmission of SARS-CoV-2 by symptomatology and post evaluation testing.

## Introduction

A cluster of viral pneumonia cases associated with a novel Coronavirus (2019-nCoV) was first identified in Wuhan, Hubei Province, China, in December 2019 and has rapidly spread around the world, causing a global health crisis.^1^ The disease was subsequently named Coronavirus Disease—2019 (COVID-19) by the World Health Organization (WHO) and has been designated SARS-CoV-2.^2^ Significant concern has arisen within the global community to the potential risks of infectious transmission of SARS-CoV-2 to the surgical team during endoscopic sinonasal and skull base surgery. As information has rapidly evolved, it has become clear that the presence of elevated viral load in the upper airway mucosa impacts not only skull base cases but also virtually all diagnostic and therapeutic intranasal procedures routinely performed by Otolaryngologists. Despite this, there has been little to no evidence-based data to guide best practices thus far. Although we hope this will be one of the first of many studies coming from our scientific community, we have attempted to evaluate the literature regarding aerosol generating procedures and evaluate a protocol to protect our patients and our colleagues in the face of this new threat.

It is important to recognize that asymptomatic COVID-19 patients may still be highly contagious. Asymptomatic adult carriers of COVID-19 have been reported,^3^ and asymptomatic infection appears to be more common in children.^4^ Thus far, there is no definitive evidence of vertical transmission from infected mothers to a fetus, although anti-SARS-CoV-2 IgM antibodies were detected in 1 infant immediately after birth.^5–7^ Although the precise mechanism of SARS-CoV-2 transmission has yet to be elucidated, the primary mode appears to be via contact, respiratory droplets, and aerosols; however, transconjunctival and fecal-oral transmission may also occur.^8^ Social distancing and isolation have therefore become one of the key methods of reduction in viral transmission. To reduce nosocomial transmission, the American Academy of Otolaryngology—Head & Neck Surgery (AAO-HNS) currently recommends limiting care to time-sensitive and emergent problems. When patient care is required, appropriate measures should be taken to prevent transmission from potentially infected patients to other patients or health-care providers.^9^

We implemented a protocol in our tertiary referral center, and after greater than 150 cases, over 5 weeks, we have succeeded in protecting our health-care providers. The objective of this study is to help define a protocol that can help the otolaryngologists and health-care staff from contracting this illness.

## Methods

We designed a prospective cohort study evaluating transmission of SARS-CoV-2 to our health-care providers at our tertiary referral center, in which we have performed a case series made up of patients from our practice and also those managed in the Emergency Department and Intensive care units over 5 weeks from March 11, 2020, through April 14, 2020, in order to determine the incidence of COVID-19 positive cases in our providers after the application of our proposed protocol. All aspects of this study were reviewed and approved by the Baton Rouge General Institutional Review Board (IRB00005439).

Our protocol adheres to Centers for Disease Control and Prevention and WHO guidelines of how to handle patients in the clinic, hospital, and operating theatre. In all settings, we implemented the use of p100 filter respirators to all providers. These masks were purchased as an alternative to powered, air-purifying respirators (PAPRs), because they are more widely available and less expensive. These masks provide for the highest personal respiratory protection as p100 respiratory filters can reliably block particles 0.3 µm or larger. Eye protection, with face shields or goggles, was implemented to prevent potential ocular transmission. We reduced the number of the health-care providers in all evaluation settings to reduce the volume of transmission. Both goggles and respirators were sterilized after use per manufacturers’ recommendations. Per state-mandated guidelines, we also reduced the number of patients by managing only those deemed as urgent and emergent, which would otherwise result in significant worsening if a delay in care were to occur. In the clinic setting, we scheduled patients at a minimum of 30-minute intervals to avoid patient-to-patient contact within the waiting area, and patients were only allowed to bring 1 companion if needed for assistance. All patients underwent date of service symptom screening questionnaire, recording of temperature, and were given a surgical mask prior to clinic entrance. All care team members underwent daily symptom screening questionnaire and recording of temperature. All examination rooms were terminally cleaned after each use while using designated personal protective equipment.

Per recommendations, our surgical cases were managed in such a way that elective cases were canceled indefinitely, until respective governmental agencies deemed appropriate. Urgent cases underwent COVID-19 testing within 24 hours prior to proceeding, while temporizing the primary ailment. Emergent or unavoidable cases proceeded in an emergent manner, and all involved personnel were provided and required to wear p100 filter respirators, eye protection along with surgical gowns and gloves (Table 1). In emergent cases where testing was pending or not available, we proceeded with the assumption of a positive test, and all involved personnel were provided and required to wear p100 filter respirators, eye protection along with surgical gowns and gloves.

**Table 1.** COVID-19: Health-care Providers Protection Protocol During Surgical Procedures.

Surgical Cases	Elective	Urgent	Emergent
COVID-19 testing	Postpone indefinitely	COVID-19 testing with 24 hours	Proceed as + COVID-19
Care team respiratory protection	NA	P100 filter^a^	P100 filter^a^
Care team eye protection	NA	Goggles^b^/Face shields	Goggles^b^/Face shields
Care team PPE	NA	Surgical gowns and gloves	Surgical gowns and gloves

Abbreviations: NA, not applicable; PPE, personal protective equipment.

^a^NORTH 7700 Series Silicone Half Mask.

^b^Category 1 personal protective equipment. Comply with EC Directive 89/686.

We also established a protocol concerning the handling the nasal endoscopy, in which we ensured the absolute necessity of its use, with no use of topical decongestant or local anesthetic solution to decrease aerosolization of particles during the examination. The use of surgical scrubs with, disposable gloves, eye protection, and p100 filter respirators along with the use of video monitoring rather than direct eye piece for visualization was required during the endoscopic examinations (Table 2). Sterilization of equipment practices was unchanged from standard procedures following each encounter.

**Table 2.** COVID-19: Health-care Providers Protection Protocol During Clinic Procedures.

Clinic Procedures	Exam	Endoscopy
Care team respiratory protection	P100 filter^a^	P100 filter^a^
Care team eye protection	None	Barrier Glasses
Care team PPE	Surgical scrubs and gloves	Surgical scrubs and gloves
Patient scheduling	30-minute intervals	30-minute intervals

Abbreviation: PPE, personal protective equipment.

^a^NORTH 7700 Series Silicone Half Mask

All health-care providers in the cohort underwent COVID-19 testing at the end of the current study via reverse transcription polymerase chain reaction nasal/nasopharyngeal swab along with point-of-care evaluation for both IgM and IgG for SARS-CoV-2.

## Results

Our implemented protocol resulted in zero transmissions in our health-care providers (*P* < .05), despite significant amounts of exposure to positive cases. Our case series of exposure consisted of 152 endoscopic sinonasal, skull base, and airway procedures, including transsphenoidal resection of pituitary adenoma for pituitary apoplexy with vision loss, modified endoscopic Lothrop procedure for an infected mucopyocele with orbital involvement, and 3 cases of sphenopalatine artery ligation for uncontrolled posterior epistaxis despite packing (positive SARS-CoV-2). We have also completed 7 cases of tracheostomy with placement of tracheostomy tube, all of which tested positive at the time of procedure, for SARS-CoV-2, for extended intubation (>2 weeks). In the clinic and hospital ward setting, we performed greater than 100 nasal endoscopies on patients who were deemed urgent or emergent. Of the 152 total cases, 26 (17.1%) patients tested positive for SARS-CoV-2, 11 (7.2%) patients tested negative for SARS-CoV-2. Moreover, 115 patients had not undergone testing at the time of the procedure due to testing availability, despite an additional 35 (23%) patients exhibiting concerning symptoms based on the symptom screening questionnaire.

Statistical analyses were performed using SPSS v 22 (SPSS Statistics for Windows, version 22.0; IBM, Armonk, New York). Descriptive data are presented as percentages and means ± standard deviation. Kendall’s tau-B was used for ordinal values. Chi-squared analysis was used for relationships of nominal variables. The prevalence of related comorbidities was also compared across treatment cohorts using Pearson chi-square analysis for relationships of nominal variables. Student’s *t* test (2-tailed) was used for comparisons of parametric data. Results were deemed significant with a *P* value of *<*.05.

## Discussion

The disease COVID-19 resulting from the novel coronavirus strain (SARS-CoV-2) represents an extraordinary threat to the health of the global population. Since its emergence in Wuhan, China, in December 2019, it has rapidly spread throughout the world following an exponential growth curve prompting it to be classified as a pandemic by the WHO on March 11, 2020. In addition to the accelerating death toll among patients, evolving information regarding infection transmission among health-care workers has raised concerns within the medical workforce regarding best practices for personal protective equipment (PPE).^10^

Anecdotal reports regarding high rates of infection specifically among Otolaryngologists^11^ have raised further critical questions with respect to the safety of performing both outpatient endoscopy as well as sinus and skull base surgery in patients during this pandemic. Otolaryngologists are at increased occupational risk for contracting COVID-19 relative to other specialties, due to high concentrations of virus in the nasal cavity, nasopharynx, and oropharynx.^12,13^

Patient use of surgical masks is impractical for the majority of Otolaryngology patient encounters, and therefore Otolaryngology providers should take appropriate personal protective measures. Concentrations of the SARS-CoV-2 virus appear to be highest in the nasopharynx and oropharynx, and therefore any patient evaluation involving examination or instrumentation of or through the oral cavity, oropharynx, nasal cavity, or nasopharynx should be considered high risk for SARS-CoV-2 exposure.^9,13^

According to Patel et al., concerns exist that N95 masks are not enough to control the spread. Not until the use of PAPRs did the spread become controlled. This article postulated guidelines to proceed dealing with cases during this crisis. All nonurgent/emergent cases are postponed. Preoperative testing was implemented, and full PAPRs were deemed necessary for all team members handling the urgent/emergent cases with pending or positive test results. In the clinic setting, Patel et al. implemented N95 masks, face shields, and gowns for all nasal endoscopies.^14^

Workman et al. postulated that the small size of the droplet particles and the extended travel of the aerosols urges for the use of PPE, with the use of N95 respirators. He also evaluated aerosol generating procedures and the effect of sneezing, in which aerosols were able to travel between 30 cm to 66 cm. This was prevented with the use of the intact and modified valved endoscopy of the nose and throat (VENT) masks. Workman et al. opted for the use of the PPE as an effective barrier technique in the outpatient clinic endoscopy, cold instrumentation in endonasal surgical procedures, even with the use of the microdebrider, as all these procedures were associated with low risk of viral aerosol transmission. They suggested that the low oscillation speeds and the continuous local suction with the microdebrider maybe associated with such low risk of aerosol transmission. In contrast, the use of high-speed drill was associated with high airflow velocities and so higher risk of droplet contamination.^10^

There are many uncertainties regarding the immediate future of the practice of rhinology as it pertains to COVID-19, but we feel that with safe protocols in place, we will be able to proceed forward with appropriate and timely care of patients in need. We recommend the use of this protocol as a guide for otolaryngologists to reduce the incidence of SARS-CoV-2 transmission. Although initial reports have advocated for the use of PAPRs, we have found that p100 respiratory filters provide appropriate respiratory protection and are more readily available and less expensive. We also recommend testing health-care providers who develop suspicious symptoms and will continue to evolve and likely expand this practice as additional testing kits become more available and accurate. Although there are great uncertainties with this pandemic, we feel that with appropriate caution and logic, with the evolution of protective protocols, we will be able to return to proper care of all patients.

## Conclusion

Our proposed protocol had succeeded in controlling the viral transmission to our health-care providers with no incidence of transmission, despite managing significant numbers of patients with actively positive SARS-CoV-2 results. In the current state of this pandemic, we do not recommend proceeding with elective evaluation or procedures per state and federal guidelines, but our results do provide evidence that with appropriate protocols for protection, we are able to safely manage patients who need intervention.
